# Chronic Kidney Disease Influences Multiple Systems: Describing the Relationship between Oxidative Stress, Inflammation, Kidney Damage, and Concomitant Disease

**DOI:** 10.1155/2015/806358

**Published:** 2015-03-15

**Authors:** Patrick S. Tucker, Aaron T. Scanlan, Vincent J. Dalbo

**Affiliations:** ^1^Clinical Biochemistry Laboratory, Central Queensland University, Bruce Highway Rockhampton, Building 81, Rockhampton, QLD 4703, Australia; ^2^Human Exercise and Training Laboratory, Central Queensland University, Bruce Highway Rockhampton, Building 81, Rockhampton, QLD 4703, Australia

## Abstract

Chronic kidney disease (CKD) is characterized by increased levels of oxidative stress and inflammation. Oxidative stress and inflammation promote renal injury via damage to molecular components of the kidney. Unfortunately, relationships between inflammation and oxidative stress are cyclical in that the inflammatory processes that exist to repair radical-mediated damage may be a source of additional free radicals, resulting in further damage to renal tissue. Oxidative stress and inflammation also have the ability to become systemic, serving to injure tissues distal to the site of original insult. This review describes select mediators in the exacerbatory relationship between oxidative stress, inflammation, and CKD. This review also discusses oxidative stress, inflammation, and CKD as they pertain to the development and progression of common CKD-associated comorbidities. Lastly, the utility of several widely accessible and cost-effective lifestyle interventions and their ability to reduce oxidative stress and inflammation are discussed and recommendations for future research are provided.

## 1. Introduction

It is estimated that 1 in 10 Australians over the age of 18 (1.7 million) have clinical evidence of chronic kidney disease (CKD) [1]. In Australia, incidence and prevalence rates of CKD are projected to increase by 29% by 2020 [2]. The increased incidence and prevalence of CKD is suggestive of an associated increase in the financial burden produced by CKD, which is projected to increase by 33% by 2020 [2]. A progressive and irreversible condition, CKD, is associated with an increased risk of developing comorbidities such as type 2 diabetes [3] and cardiovascular disease (CVD) [4]. Although it is possible to slow the progression of CKD during its early stages, CKD-related risk factors (e.g., hyperglycemia and hypertension) and comorbidities become less manageable as CKD inevitably progresses, resulting in a life expectancy that decreases in parallel with decreasing kidney function [5]. Considering that CKD is irreversible, CKD-related therapies that focus on reducing factors which exacerbate disease progression are ideal. Furthermore, slowing CKD progression may delay the development and progression of life-shortening comorbidities [4, 6, 7]. As such, this review will focus on the mechanisms by which oxidative stress and inflammation influence the development and progression of CKD, as well as the development and progression of CKD-associated comorbidities. In addition, this review discusses the utility of several widely accessible and cost-effective lifestyle interventions and their ability to reduce oxidative stress and inflammation, as well as recommendations for future research.

## 2. Oxidative Stress and Inflammation: Key Mediators in CKD

Emerging evidence suggests that reducing oxidative stress and inflammation are two of the most apposite approaches, in terms of slowing CKD progression. Studies have demonstrated that markers of oxidative stress and inflammation are significantly elevated in CKD patients, relative to healthy counterparts [8, 9]. Moreover, oxidative stress, as measured by 8-isoprostaglandin F_2_alfa (*r* = −0.68, *P* < 0.01) [6], and inflammation, as measured by neopterin (*r* = −0.32, *P* < 0.01) [10], have a significant inverse relationship with estimated glomerular filtration rate (eGFR), the most commonly consulted marker of kidney function. Despite their well-established relationship, intricate interactions between oxidative stress, inflammation, and renal damage make it difficult to discern which process is primarily responsible for initiating the series of events that eventually lead to kidney failure.

## 3. Linking Oxidative Stress and Inflammation to CKD Progression

**Figure 1:**
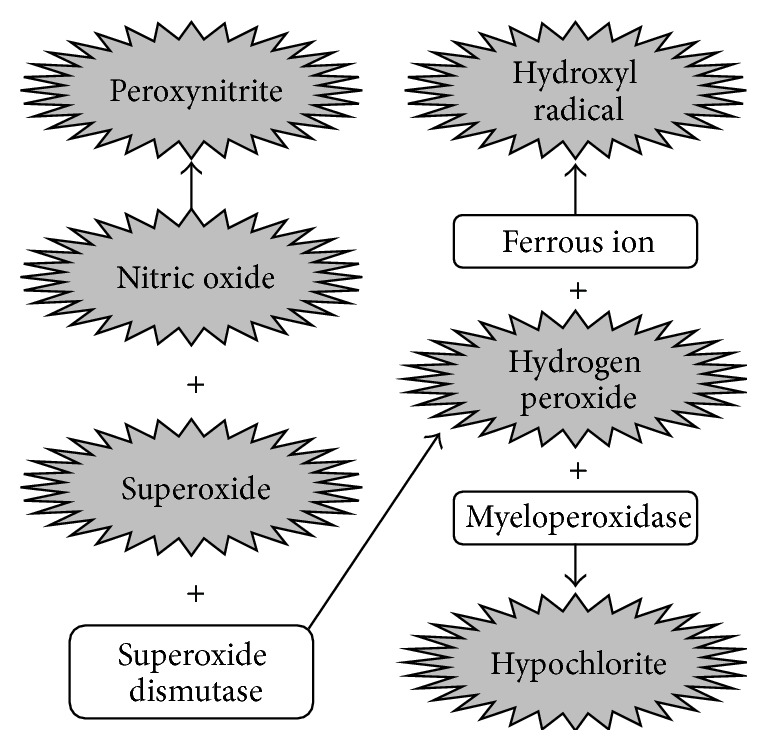
Radical reactions leading to initial renal injury. Intermediates in grey are harmful to biological molecules. Compounds in white, although not directly damaging, are involved in harmful reactions.

The primary pathological mechanism that links oxidative stress, inflammation, and CKD progression is characterized by an initial injury in the kidney due to the activities of intra- and extracellular oxygen-derived radicals and the resultant inflammatory response. Radicals such as superoxide and hydroxyl radical readily interact with the molecular components of a nephron [11]. Several reviews have described radical-molecule interactions, including the oxidation of amino acids resulting in the loss of important functional properties [12, 13], lipid peroxidation of cell membranes resulting in decreased membrane viability [12, 14], and cleavage and crosslinking of renal DNA resulting in harmful mutations [15, 16]. Radical interactions of this sort result in immediate damage to the nephron and the production of secondary radicals. For example, superoxide may be converted to hydrogen peroxide via superoxide dismutase, which may be further converted into hypochlorite, via myeloperoxidase, or hydroxyl radical, via Ferrous ion. Superoxide radical may also interact with nitric oxide to form peroxynitrite [17]. These secondary radicals bear the same destructive potential as initiating radicals [17], leading to a deleterious chain reaction characterized by cellular/molecular-level nephron damage and continued radical production [7] (Figure 1).

**Figure 2:**
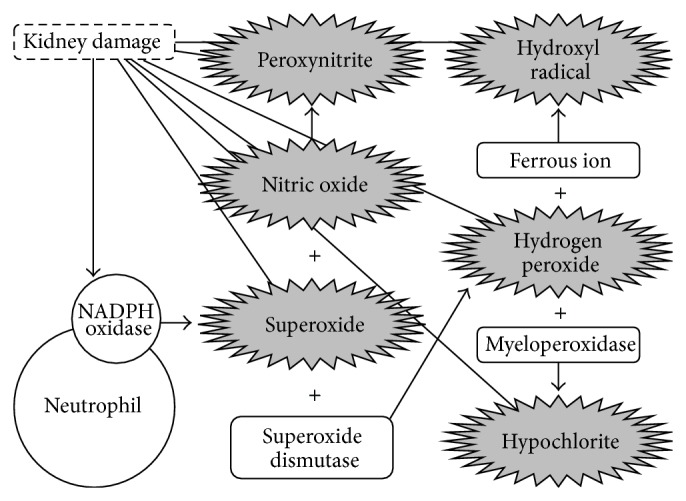
Interactions between radical damage, inflammation, and renal injury. Intermediates in grey initiate damage of the molecular components of renal tissue. Compounds in white, although not directly damaging, are involved in harmful reactions. Damage resulting from intermediates in grey promotes an inflammatory response during which additional superoxide is released via phagocytic NADPH oxidase activity.

As radical-mediated nephron damage occurs, the resultant inflammatory response, which normally serves as a protective and reparative mechanism, stimulates the formation of additional free radicals [18]. Neutrophils (and other phagocytes) recruited to the damaged nephron produce superoxide via their membrane-associated nicotinamide adenine dinucleotide phosphate (NADPH) oxidase system, in which electrons are transferred from NADPH inside the cell across the membrane and coupled to molecular oxygen, resulting in superoxide [18]. Superoxide and other radicals, as well as their modified targets, continue to promote kidney-specific injury or act as messenger molecules, resulting in a locally (i.e., renal) sustained inflammatory response [7]. This radical-induced immune response encourages the release of additional proinflammatory signals which inevitably result in the formation of additional radicals and/or reactive oxygen species (ROS) and continued damage to the molecular components of nephrons [7]. Following prolonged insult (i.e., recurrent oxidative stress and chronic inflammation), radical-mediated damage eventually results in nephron degradation so extensive that tissue/organ damage becomes apparent (e.g., reduced eGFR) (Figure 2) [9].

In the context of tissue/organ damage, kidney injury molecule-1 (KIM-1) has emerged as a useful biomarker; KIM-1 is almost exclusively expressed in renal tissue [19] and is absent in healthy kidneys [20, 21]. An epithelial cell adhesion molecule, KIM-1, is expressed in damaged tubular epithelial cells undergoing dedifferentiation and proliferation [21]. Normally undetectable [20, 21], KIM-1 expression is markedly increased following radical-mediated renal injury and is associated with glomerular influx of inflammation-mediated macrophages [22]. Evidence suggests that KIM-1 may phagocytose apoptotic and necrotic cells in the tubule of the kidney, helping to clear the lumen of the epithelial tubule of cellular debris, thereby reducing intratubular obstruction [23]. KIM-1-mediated phagocytosis of apoptotic cells may also influence the generation of anti-inflammatory cytokines. An important renal repair molecule, hepatocyte growth factor has been shown to be upregulated in epithelial cells that have phagocytosed apoptotic cells [24]. Thus, it appears that KIM-1 contributes to the clearance of cellular debris and influences an important regenerative mechanism, resulting in improved epithelial health. This may explain why, in acute instances, KIM-1 appears to adopt a protective role. However, in scenarios characterized by chronic insult, KIM-1 expression becomes deleterious, serving as a marker and promoter of kidney damage [25].

One study reported that tubules expressing KIM-1 regulated the response of inflammatory cells through the secretion of chemokines and cytokines, suggesting that KIM-1-expressing epithelial cells play a role in the pathogenesis of tubulointerstitial inflammation during chronic renal injury [26]. Furthermore, tubules expressing KIM-1 exhibit high proliferative activity and have characteristics similar to myofibroblasts, suggesting that KIM-1 expression is associated with dedifferentiation of epithelial cells and the development of tubulointerstitial fibrosis [25]. Injuries of this sort further aggravate already damaged renal tissue, giving rise to additional radicals and increased inflammation. Left unabated, renal oxidative stress and inflammation may promote injury in distal tissues due to their potential to become systemic [18].

## 4. Linking Oxidative Stress, Inflammation, and CKD Progression to Comorbidities

In addition to renal effects, oxidative stress and inflammation offer the primary explanation as to why patients with CKD are likely to experience concomitant type 2 diabetes and/or CVD. Proinflammatory cytokines, such as tumour necrosis factor-*α* (TNF*α*), initiate proinflammatory signals by binding to TNF*α* receptors, TNFR1 and TNFR2, on tubular (and other) cell surfaces, triggering signalling pathways that activate nuclear factor *κ*B (NF*κ*B) transcription factors [27, 28]. Normally bound to an inhibitor protein (I*κ*B), NF*κ*B can exacerbate in-progress inflammatory responses [28]. Specifically, the presence of free radicals encourages the degradation of I*κ*B, allowing the translocation of NF*κ*B dimers to the nucleus. The translocation of NF*κ*B dimmers to the nucleus prompts the transcription of genes involved in systemic inflammatory responses, thereby encouraging downstream generation of free radicals via phagocytic activity (often referred to as “respiratory bursts” or “oxidative bursts”) [28].

Recent evidence suggests that phospholipase C epsilon 1 (PLC*ε*1) can also regulate NF*κ*B activity [29]. PLC*ε*1 is implicated in CKD as* PLCε1* gene mutations have been associated with early onset nephrotic syndrome [30], proteinuria [30], mesangial sclerosis [31], and glomerulosclerosis [30, 31]. Nevertheless, little is known about the direct relationship between PLC*ε*1 expression and kidney damage. What is known is that PLC*ε*1 catalyses the hydrolysis of phosphatidylinositol, 4,5-bisphosphate (PIP_2_), generating second messengers inositol 1,4,5-trisphosphate (IP_3_) and diacylglycerol (DAG), leading to protein kinase C (PKC) activation and subsequent proinflammatory responses via PKC-mediated activation of NF*κ*B [32, 33].

Considering this, TNFR1, TNFR2, and PLC*ε*1 are important therapeutic targets as blocking NF*κ*B activation helps to reduce downstream systemic inflammation as well as the inflammation-mediated generation of free radicals [28, 29]. The inhibition of NF*κ*B activation is partially responsible for the positive effects of endogenous antioxidant enzymes superoxide dismutase (SOD), glutathione peroxidase (GPX), and catalase (CAT), which serve to quench free radicals while simultaneously inhibiting the nuclear translocation of NF*κ*B [34, 35], thereby reducing further systemic inflammation and radical generation.

**Figure 3:**
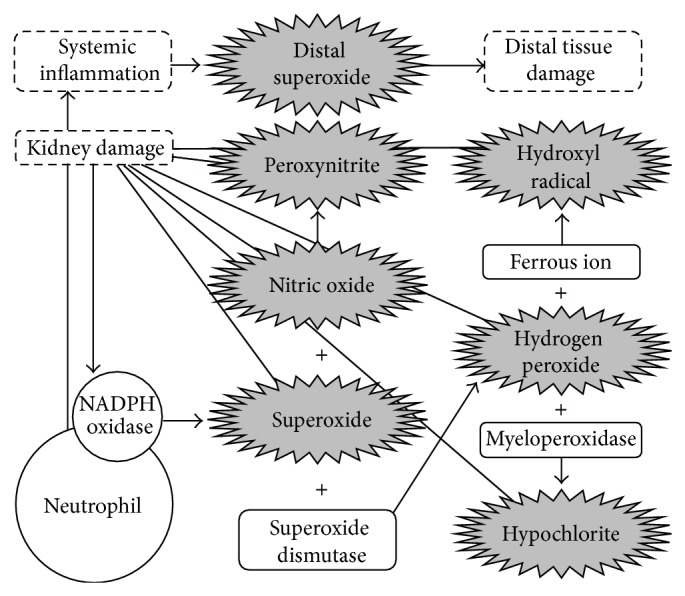
Interactions between radical damage, inflammation, and distal injury. Intermediates in grey initiate damage of the molecular components of renal tissue. Compounds in white, although not directly damaging, are involved in harmful reactions. Damage resulting from intermediates in grey promotes an inflammatory response during which additional superoxide is released via phagocytic NADPH oxidase activity. If sustained, this process may lead to a systemic inflammatory response that can result in damage to tissues that are distal to the kidney, such as the pancreas (type 2 diabetes) and vasculature (cardiovascular disease), via related increases in the production of reactive oxygen species (ROS). For example, the transcription of NF-*κ*B-dependent genes may* regulate* levels of cellular ROS; the NF-*κ*B pathway may be activated by stimulation of proinflammatory receptors, such as the TNF receptor superfamily. In turn, NF-*κ*B activation may also be* regulated by* cellular levels of ROS; ROS can activate NF-*κ*B through alternative I*κ*B phosphorylation, resulting in the degradation of I*κ*B.

The inability to suppress continual inflammatory responses and radical formation results in systemic effects that are characterized by damage to tissues distal to the site of the original injury (Figure 3). During oxidative stress, endothelial cells upregulate the expression of adhesion molecules [36, 37], allowing leukocytes (typically neutrophils) to adhere to this new (distal) site of injury. These neutrophils transmigrate into the intima of an artery and promote the accumulation of monocytes at the injured site via neutrophil-derived cathelicidin binding at the luminal surface of the arterial endothelium [38, 39]. Monocytes bind cathelicidin and transform into foam cells which may develop into atherosclerotic lesions, the most common manifestation of CVD following hypertension [39]. The immune response plays a role in the formation of atherosclerotic lesions and is responsible for the generation of additional circulating free radicals which have the ability to damage additional tissues [18].

Insulin producing *β*-cells of the pancreas are especially sensitive to free radicals [40, 41] due to their low expression of the antioxidant enzymes SOD, GPX, and CAT [42]. Upregulated activity of SOD [43], GPX [44], and CAT [43] is associated with decreased *β*-cell damage, indicating that these enzymes play an important, albeit indirect, role in glucose regulation [45, 46]. However, low local concentrations of SOD, GPX, and CAT leave *β*-cells prone to attack by free radicals, resulting in permanently decreased insulin production which leads to hyperglycemia [44, 47]. Hyperglycemia, in turn, leads to further local (pancreatic) and systemic damage via increased production of mitochondrial ROS [48], nonenzymatic glycation of proteins [49], glucose autoxidation [50], and the activation of stress-sensitive signalling pathways such as the NF*κ*B signalling pathway [51, 52]. Fortunately, there are means by which this detrimental sequence of events can be interrupted, allowing an opportunity for tissue repair, as well as a reduction in additional damage.

## 5. Ameliorating Oxidative Stress- and Inflammation-Mediated Damage in CKD

The association between oxidative stress [53], inflammation [54], and CKD progression is well established. Reviewed in detail elsewhere, researchers have identified a number of important biomarkers that influence CKD progression as well as help inform clinicians and researchers as to the disease status of CKD patients [53]. However, it is important to note that interactions between oxidative stress, inflammation, and CKD progression are cyclical, with no distinct initiator or terminator. Nevertheless, there are therapeutic options available that help to discourage the deleterious cycle that ultimately results in decreased renal function. Although the relationship is often indirect, lifestyle interventions such as exercise, structured diet, and weight loss act to decrease the damage caused by an overabundance of free radicals and sustained inflammatory processes.

Chronic aerobic exercise training (AET) has emerged as a promising therapy in terms of reducing injury stemming from oxidative stress and inflammation. In CKD patients, the therapeutic efficacy of chronic AET has been well established [55–58]. Several explanations regarding chronic AET-mediated benefit exist. The primary mechanism appears to be a chronic AET-induced upregulation of SOD [59], GPX [60], and CAT [61]. There is also evidence to suggest that chronic AET leads to reduced mitochondrial ROS [62], reduced expression of ROS-generating enzymes NADPH oxidase [40] and xanthine oxidase [63], and a downregulation of mitochondrial monoamine oxidase-A [64], a major source of oxidative stress via hydrogen peroxide generation. To date, the ability of chronic AET to reduce oxidative stress and inflammation, specifically in patients with CKD, lacks strong evidence [56]. However, this is seemingly due to a lack of investigations that carefully examine the effects of chronic AET on oxidative stress and inflammation in CKD. Traditionally, studies examining chronic AET and CKD have favoured clinical (e.g., eGFR) and functional (e.g., peak VO_2_) markers.

Dietary interventions have also been shown to be an effective strategy in terms of reducing oxidative and inflammatory damage. Reviewed elsewhere [65], the nutritional status of CKD patients is highly influential in terms of CKD progression and overall health status. Poor nutritional status is more prevalent in CKD patients, compared to healthy counterparts, resulting in related reductions in antioxidant status [66] and increases in oxidative stress [65]. Moreover, because many patients with CKD adhere to a reduced-protein diet (on average: 0.6 g/kg/day versus 1.0 g/kg/day in healthy persons) [67], CKD patients may be deficient in micronutrients with antioxidant effects such as zinc [68] and certain amino acids such as cysteine [65, 69]. Considering this, diet-related considerations should be carefully evaluated in CKD patients. As such, several compelling reviews have been written on the topic [70–73].

Interestingly, evidence suggests that calorically restricted diets significantly reduce lipid peroxidation as measured by malondialdehyde-thiobarbituric acid [74] and oxidized low-density lipoprotein [75], as well as inducing NAD-dependent deacetylase sirtuin-3, mitochondrial (SIRT3) which acts to reduce cellular ROS and promote stress resistance by deacetylating superoxide dismutase 2, mitochondrial (SOD2) [76]. However, it is important to note that health-related benefits stemming from a dietary intervention may be mediated by accompanying weight loss and this positive effect may be further increased by the addition of chronic AET [77]. For instance, important CKD-related markers, serum creatinine and albumin, were significantly improved following dietary weight loss in men and women with CKD-related risk factors, despite the fact that these patients had not been formally diagnosed with CKD [77]. This beneficial effect was more pronounced in participants who also underwent chronic AET, a combined intervention that resulted in significant improvements to eGFR and reductions in c-reactive protein [77]. Results from this study suggest that combination therapy (diet and chronic AET) may be more beneficial in terms of reducing the oxidative and inflammatory damage apparent in early stage CKD, relative to a single-therapy approach. Despite these encouraging findings, very few studies have examined the utility of combination therapy (diet and chronic AET) in patients with diagnosed CKD [78, 79].

## 6. Summary and Future Research

Chronic kidney disease is characterized by an increase in oxidative stress and inflammation. Increased oxidative stress and inflammation may serve to promote additional damage to the kidney, as well as initial or additional damage to distal tissues, resulting in the development or progression of concomitant diseases. In this regard, it becomes apparent that reducing oxidative stress and inflammation is imperative as damage mediated by oxidative stress and inflammation is cyclical and potentially systemic, serving to injure local tissue, as well as tissue distal to the site of original insult.

Future research should focus on interventions that aim to reduce oxidative stress and inflammation in patients experiencing various stages of CKD. Several lifestyle interventions [62, 76, 77] exist that help to directly reduce oxidative stress and inflammation and indirectly reduce these processes by improving related risk factors such as eGFR [77], blood pressure [4], and glucose regulation [48]. Nevertheless, the mechanisms by which these benefits are achieved have yet to be fully described. Furthermore, intervention studies do not comprehensively address the various stages of CKD. This is noteworthy as each stage of CKD is characterized by differing levels of oxidative stress [9] and inflammation [8, 9], as well as varying levels of risk in regard to the development of comorbidities such as type 2 diabetes [52] and CVD [5].

Oxidative stress [9] and inflammation [8] influence the development and subsequent progression of CKD. Moreover, oxidative stress and inflammation are the primary reasons why CKD is often accompanied by comorbidities such as type 2 diabetes [80] and CVD [6]. Due to the potentially self-exacerbating and cyclical nature of oxidative stress and inflammation, diseases characterized by these two risk factors (diseases such as CKD, type 2 diabetes, and CVD) are necessarily intertwined in that the progression of one disease may lead to the development or progression of another [11]. In terms of cost-effective and easily accessible interventions, a combination of diet and chronic AET may be the most beneficial as diet and chronic AET independently have the ability to directly reduce oxidative stress [59, 62] and inflammation [63, 77], while simultaneously influencing secondary sources of oxidative stress and inflammation (e.g., hyperglycemia and hypertension). However, few studies have examined the utility of combined diet and chronic AET in patients with CKD [77, 79]. Considering the multifaceted means by which diet and chronic AET help to reduce oxidative stress and inflammation, combined with their accessibility and cost-effectiveness, it stands to reason that a combined diet chronic AET approach may prove more beneficial for patients with varying degrees of renal impairment, relative to diet or chronic AET alone [64, 77, 78]. Investigations that examine the combined efficacy of dietary and chronic AET interventions in patients with CKD, as well as the mechanisms by which efficacy is obtained, are strongly encouraged.

## Abbreviations

AET: Aerobic exercise training
CAT: Catalase
CKD: Chronic kidney disease
CVD: Cardiovascular disease
DAG: Diacylglycerol
eGFR: Estimated glomerular filtration rate
GPX: Glutathione peroxidase
IP3: Inositol 1,4,5-trisphosphate
I*κ*B: Nuclear factor of kappa light polypeptide gene enhancer in B-cells inhibitor
KIM-1: Kidney injury molecule-1
NADPH: Nicotinamide adenine dinucleotide phosphate
NF*κ*B: Nuclear factor kappa B
PIP2: Phosphatidylinositol, 4,5-bisphosphate
PKC: Protein kinase C
PLC*ε*1: Phospholipase C epsilon 1
ROS: Reactive oxygen species
SIRT3: Deacetylase sirtuin-3, mitochondrial
SOD: Superoxide dismutase
SOD2: Superoxide dismutase 2, mitochondrial
TNF*α*: Tumour necrosis factor alpha.
